# DNA Metabarcoding Unveils Habitat-Linked Dietary Variation in Aerial Insectivorous Birds

**DOI:** 10.3390/ani15070974

**Published:** 2025-03-27

**Authors:** Fatihah Najihah Arazmi, Nor Adibah Ismail, Ummi Nur Syafiqah Daud, Mohammad Saiful Mansor

**Affiliations:** 1 Department of Biological Sciences and Biotechnology, Faculty of Science and Technology, Universiti Kebangsaan Malaysia, Bangi 43600 UKM, Selangor, Malaysia; n.fatihahnajihah@gmail.com; 2 Department of Earth Sciences and Environment, Faculty of Science and Technology, Universiti Kebangsaan Malaysia, Bangi 43600 UKM, Selangor, Malaysia; nadibahismail@gmail.com (N.A.I.); ummisyafiqah96@gmail.com (U.N.S.D.)

**Keywords:** aerial insectivores, agricultural, diet, oil palm, paddy field

## Abstract

The conversion of tropical forests into urban and agriculture landscapes may alter insect populations through habitat disturbance and impact the diets of aerial insectivores. Most dietary studies on aerial insectivores have limitation on identifying prey at higher taxonomic levels in broad landscapes, restricting species-level identification and thus making a detailed dietary comparison impossible. This study examines the dietary changes through adaptation of house-farm swiftlets (*Aerodramus* sp.) and Pacific swallows (*Hirundo tahitica*) across three distinct habitats in Peninsular Malaysia: mixed-use landscapes, oil palm plantations, and paddy fields. High-throughput DNA metabarcoding with ANML primers targeting mitochondrial *CO1* gene, identified 245 arthropod prey species, with six dominant orders: Coleoptera, Diptera, Blattodea, Hemiptera, Hymenoptera, and Lepidoptera. Mixed-use landscapes supported the highest dietary diversity and niche breadth, reflecting their ecological complexity. Paddy fields exhibited moderate diversity, while oil palm plantations demonstrated the lowest diversity, influenced by simplified vegetation structures and limited prey availability. The consumption of agricultural pests and vector species highlights the critical ecological role of aerial insectivorous birds in natural pest management and mitigating vector-borne disease risks. This research emphasizes the importance of conserving habitat heterogeneity to sustain the ecological services provided by these birds, benefiting both agricultural productivity and public health.

## Simple Summary

Aerial insectivorous birds, such as swiftlets and swallows, play a vital role in maintaining ecosystem balance by regulating insect populations, including agricultural pests. However, studying their diet is challenging, as they feed in flight and consume prey too small for direct observation. To address this, we employed high-throughput DNA metabarcoding to analyse DNA fragments from bird droppings, revealing a diverse diet that includes agricultural pests and disease-carrying species. Notably, birds in mixed-use landscapes exhibited the highest dietary diversity, whereas those in oil palm plantations had the least. These findings highlight the importance of aerial insectivorous birds in natural pest control and emphasize the need to conserve heterogeneous landscapes to sustain their populations and the ecological services they provide.

## 1. Introduction

Aerial insectivorous birds exhibit extensive distribution across habitat types, including forests, agricultural lands, open spaces and urban landscapes. Their adaptability allows them to thrive in various ecosystems, primarily because of the abundance of flying arthropod prey across diverse habitats. Conversion of tropical forests into urban and agricultural landscapes in many developing countries has led to the local and regional extinction of numerous arthropods [1]. These extinctions may affect the stability of ecosystems and cause major alterations in the diets of aerial insectivorous birds. Growing urbanisation has led to the separation of green areas by impermeable surfaces, which has led to the creation of more fragmented green patches, decreased patch connectivity and promoted edge habitats, which could affect the abundance and diversity of native species of arthropods [2,3,4]. Since many arthropods are sensitive to the microclimate, the community composition may affect the populations of aerial insectivorous birds that rely on arthropods as their primary prey. The main risks to insects include factors associated with agricultural intensification, such as habitat loss, temporal and spatial homogenisation, and the use of synthetic fertilisers, herbicides and pesticides [5]. 

Agricultural expansion and deforestation in Peninsular Malaysia have replaced large forest areas with crops like oil palm, rubber and paddy, leading to environmental changes and insect declines [6,7]. Malaysia, the second-largest global palm oil producer after Indonesia, contributes 26% of global production, with major plantations in Johor, Pahang and Perak [8,9]. Rubber is the second-largest crop, covering 32% of agricultural land and producing 42,554 tons in 2020 [10,11]. Malaysia also dedicates 671,679 hectares to rice cultivation, contributing 0.36% of global rice production, with key paddy fields located in Kedah, Perak, Selangor and the Kelantan Delta [12,13].

Aerial insectivorous birds are the most significantly influenced by landscape changes, and such changes can lead to a reduction in aerial insectivorous birds that mainly forage on the flying insects above the canopy layers [14]. A previous study [15] examined the challenges facing aerial insectivorous birds in modern agricultural landscapes, highlighting how declines in insect populations are closely linked to reductions in swift populations. The study emphasizes the critical association between insect availability and the viability of swift populations within these altered environments. 

Aerial insectivorous birds, such as swallows and swiftlets, are opportunistic feeders with broad diets, relying on seasonally available arthropods. They forage in open areas or above vegetation during the day. Understanding their prey preferences in different habitats is crucial, given their economic and ecological roles, especially as biological pest controls in agricultural landscapes [16,17]. Although aerial insectivorous birds are abundant and consume large quantities of insects, regulating insect populations across ecosystems [18], there is limited research comparing their diets between habitats with identification of prey at the species level.

High-throughput amplicon sequencing, particularly using ANML primer sets targeting the mitochondrial cytochrome oxidase C subunit 1 (COI) gene, has proven effective in accurately identifying dietary prey at a finer taxonomic resolution [19,20,21]. This approach enables the detection of a broader range of invertebrate taxa, improving our understanding of aerial insectivores’ diets compared to traditional morphological analysis. Previous studies have successfully applied this approach to aerial insectivores, revealing complex dietary patterns and species-specific prey preferences [22,23].

In Peninsular Malaysia, existing research on aerial insectivores’ diets has limitations. A previous study [24] examined swiftlet diets within three landscapes, identifying prey mostly at the order level, which hinders the classification of prey as potential agricultural pests [25], and focused on aerial insectivore diets within a single forest habitat, providing limited insight into dietary patterns among different ecosystems. Therefore, the present study aimed to investigate the dietary composition of aerial insectivorous birds, highlighting dietary variations between mixed-use landscapes, oil palm plantations, and paddy fields in Peninsular Malaysia, where both the Edible-nest swiftlet *Aerodramus* sp. and the Pacific swallow *Hirundo tahitica* are found. Aerial insectivorous birds also play a crucial role as biological control agents by feeding on insect pests, thereby supporting sustainable pest management in agricultural landscapes. Their predation on pest species contributes to ecosystem services, reducing the need for chemical pesticides. We also would like to emphasize the effectiveness of aerial insectivorous birds as biological control agents in agriculture landscapes. We utilised ANML primer sets, targeting the mitochondrial cytochrome oxidase C subunit 1 (*COI*) gene, for high-throughput amplicon sequencing [19] to identify key insect species contributing to dietary differences among habitats and their ecological implications for aerial insectivore feeding strategies.

## 2. Materials and Methods

### 2.1. Study Sites

**Figure 1:**
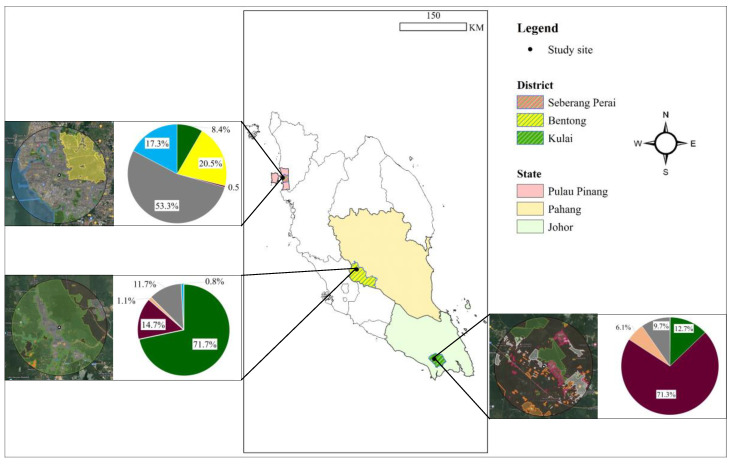
Study sites representing three selected habitats: Bentong, Pahang (mixed-use), Kulai, Johor (oil palm plantation), and Seberang Perai, Pulau Pinang (paddy field) in Peninsular Malaysia, examined for the diet composition of two aerial insectivorous bird species. The inserted pie charts illustrate the percentage of habitat types surrounding the swiftlet house-farm buildings in each study site, highlighting the dominance of specific land-use categories such as mixed-use, oil palm, and paddy field within the respective regions. (Blue: water bodies; green: forest; grey: urban settlements; orange: other plantations; purple: oil palm plantations; yellow: paddy fields).

We investigated the dietary composition and ecological interactions of two species of aerial insectivorous birds in three distinct habitat types within three states of Peninsular Malaysia: Bentong, Pahang; Seberang Perai, Pulau Pinang; and Kulai, Johor. (1) Bentong, situated 60–70 km east of Kuala Lumpur in central Peninsular Malaysia, is located in the western part of Pahang, near the Titiwangsa mountain range; (2) Kulai is situated in the southern part of Peninsular Malaysia, within the state of Johor, 270–280 km from Kuala Lumpur; and (3) Seberang Perai is located on the mainland part of Pulau Pinang and 300–320 km northwest of Kuala Lumpur (Figure 1). These three regions represent critical land-use categories that influence biodiversity and species behaviour of aerial insectivorous birds in Peninsular Malaysia. They have been selected because of the abundance and diversity of the study species, and the sites were surrounded by swiftlet house-farms identified as roosting and nesting sites for swallows and swiftlets where they could be found roosting on utility wires, building rooftops and under bridges near to the swiftlet house-farms [26,27,28]. Kulai and Seberang Perai were selected as agriculture landscapes in which oil palm plantations and paddy fields are the most dominant agriculture landscapes and occupy large areas of crop production. Bentong features a diverse landscape comprising different vegetation types, including forested areas and a variety of agricultural crops such as durian, coconut, rubber plantations, and oil palm [29]. 

To characterise the landscape profiles for each study site, Google My Maps (https://www.google.com/maps/d/, accessed on 1 February 2022) was used up to 6 km from the swiftlet house-farm for each site, considering the distance range as the average flying distance of swallows and swiftlets for foraging [30]. The landscape profiles show distinct landscape types at the three study sites. Bentong, Pahang, features a mixed-use landscape of 72% forest, 15% oil palm plantations, 12% human settlements, and 1.08% agricultural crops (e.g., durian, coconut, rubber). Kulai, Johor, is dominated by oil palm plantations (71%) with smaller areas of forest patches (13%), human settlements (19%), and other plantations (6.10%). In Seberang Perai, Pulau Pinang, the landscape is primarily urban (53%) with paddy fields (20%) contributing to seasonal arthropod prey availability, alongside water bodies (17.30%) and minor forest cover (8%). The landscape profiles confirm the three study sites consist of different landscape types: mixed-use urban-rural landscapes in Bentong, Pahang; oil palm-dominated landscape in Kampung Baru Air Bemban, Kulai, Johor; and paddy fields in Kampung Masjid Timah, Seberang Perai, Pulau Pinang (Figure 1).

### 2.2. Sample Collection

Fresh faecal samples were collected in three different regions in Peninsular Malaysia from March until October 2022. The faecal samples of the Edible-nest swiftlet were collected from the swiftlet house-farm, which is a man-made structure designed to attract Edible-nest swiftlets for the purpose of harvesting their nests. In this study, we refer to the Edible-nest swiftlet as *Aerodramus* sp. due to ongoing taxonomic uncertainties associated with swiftlets inhabiting house-farms in Malaysia. Cranbrook et al. [31] and Goh et al. [32] documented significant genetic and morphological variation among house-farm swiftlets, suggesting that they may represent a complex of closely related taxa rather than a single species. For the Pacific swallow, faecal samples were collected beneath their nesting areas and under their roosting sites. For both species, the methods of sample collection were the same. Plastic sheets measuring 20 inches × 30 inches were placed under their roosting and breeding sites. Faecal samples were swiftly obtained from the plastic sheets using sterilised forceps to prevent cross-contamination between the environments and were kept in 2 mL tubes containing 100% ethanol. All the faecal samples for both species were obtained individually, with each dropping representing a single individual from each study species. At the sites, the faecal samples were preserved in a cool box prior to being placed in a freezer at −20 °C until DNA extraction.

### 2.3. DNA Extraction, Amplification and Sequencing

The genomic DNA was extracted individually from a total of 90 faecal samples collected across three different habitats, using QIAmp DNA Stool Mini Kit (QIAGEN, Hilden, Germany), following the manufacturer’s protocol. These samples comprised 15 from Edible-nest swiftlets and 15 from Pacific swallows, representing each habitat equally. The extracted DNA was eluted in 60 µL buffer and preserved at −20 °C for subsequent analyses. PCR amplification targeted a 180-bp segment of the mitochondrial cytochrome c oxidase subunit 1 (*CO1*) gene using the ANML primer using the forward primer LCO1490 (5’-GGTCAACAAATCATAAAGATATTGG-3’) and reverse primer CO1-CFMRa (5’-GGWACTAATCAATTTCCAAATCC-3’) [19]. Individual samples were amplified using a 25 µL PCR reaction mixture comprising 12.5 µL NEXpro^TM^ e PCR 2× MasterMix Solution (Genesystems, Seoul, Republic of Korea), 8.5 µL nuclease-free water, 1.0 µL of each primer (10 µM), and 2 µL template DNA. The PCR protocol included an initial denaturation at 94 °C for 60 s, followed by two amplification phases: the first phase involved five cycles of 94 °C for 60 s, 45 °C for 90 s, and 72 °C for 90 s; the second phase included 35 cycles of 94 °C for 60 s, 50 °C for 90 s, and 72 °C for 60 s. A final extension step was performed at 72 °C for 10 min [33]. PCR products were assessed by agarose gel electrophoresis to confirm the presence of DNA fragments. Sequencing was conducted on an Illumina MiSeq platform (2 × 250 bp, Illumina, San Diego, CA, USA) following standard protocols.

### 2.4. Bioinformatics and Data Analysis

The raw sequence data in FASTQ format was processed using the QIIME 2 platform [34]. The sequencing data, consisting of both forward and reverse reads, was processed using QIIME 2 software version 22.04.3. Prior to any further analysis, a quality assessment of all raw sequences was conducted using FastQC version 0.12.0 to ensure they were suitable for the subsequent steps [35]. The initial analysis phase involved demultiplexing the sequence reads using the demux-paired function from the Cutadapt plugin. This step identified the barcode sequences assigned during sequencing, ensuring the correct allocation of reads to their respective samples. Following demultiplexing, the sequences were trimmed with the trim-paired function from Fastp to remove barcodes and primers from each sample sequence. Next, paired-end reads were merged using the Fastp function, combining overlapping forward and reverse sequences into single-end reads. This merging step optimised the data for downstream applications that perform more efficiently with single-end reads. After merging, the sequences were denoised and dereplicated, with chimeric sequences filtered out using the denoise-paired function in DADA2 22.04.3.

Subsequently, representative sequences, along with an abundance table and a feature table, were generated using the Feature-table plugin in QIIME2. The functions “feature-table_summarize” and “feature-table_tabulate-seqs” assigned unique feature IDs to each representative sequence, grouping them into distinct amplicon sequence variants (ASVs). The taxonomic classification of these ASVs was performed by comparing them against the NCBI GenBank database (https://blast.ncbi.nlm.nih.gov/Blast.cgi, accessed on 1 February 2022). Sequences showing a minimum 98% similarity were classified at the species level, those with 95% similarity at the genus level, and sequences with 90% similarity at the family level, following the methods of Mansor et al. [28].

The taxa plugin in QIIME2 was then used to develop a dietary dataset based on the taxonomic identifications obtained from the Feature-table plugin. This involved tracking occurrences in individual samples and generating multiple metrics to summarise dietary preferences between bird species. The frequency of occurrence (FOO) was calculated to reflect how often different prey items appeared across the five replicate samples. Relative read abundance (RRA) was employed as an alternative metric to estimate the proportion of prey items in the diet of aerial insectivores, representing the relative biomass consumed. Detailed calculations for these metrics are available in the Supplementary Material (Files S1 and S2).

To evaluate the variation in dietary composition between species, a permutational analysis of variance (two-way PERMANOVA) was employed on the distance matrix. This analysis utilised “Order, Family” and “Group” as predictor variables, with 9999 permutations applied to enhance the statistical robustness. These calculations used the diversity function within the PAST software (version 4.03) to ensure an accurate assessment of the abundance and diversity patterns of arthropod prey in their dietary analysis [36]. The Pianka Index (Ojk) and Levins Index (B) were calculated using R software version 4.2.1 to assess dietary overlap and niche breadth among habitats. The Pianka Index was employed to measure dietary overlap among habitats, quantifying the extent to which diets overlapped across all habitats. Additionally, the Levins Index was utilised to evaluate dietary niche breadth, reflecting the range of prey diversity consumed by aerial insectivorous birds in each habitat, with broader values indicating greater dietary adaptability and ecological resource use.

Four visualisations were generated using R to illustrate the study results [37,38]. A heatmap illustrates the relative reads abundance (RRA) of several prey species consumed by the two bird species in three different habitats. A bubble plot was generated to visualise FOO by each taxon. RRA values ≤ 0.01 were discarded from the diagram for ease of understanding. Also, a bipartite network analysis was generated to represent interactions between birds and their prey, highlighting dietary overlaps and interactions between the three different habitats. An Alluvial diagram was created to represent the correlation of dominant insect species across the three habitats.

## 3. Results

### 3.1. Sequencing Output

High-throughput metabarcoding produced 3,407,322 raw sequences from the faecal samples of Edible-nest swiftlets and Pacific swallows across the three habitats. After filtering, denoising, merging, and removing chimeras, 1,817,300 sequences remained. From these, 1833 unique ASVs were identified through CO1 gene analysis. Taxonomic identification revealed three classes, 19 orders, 230 families, 279 genera, and 245 species of arthropods in the birds’ diets across the three habitats. 

**Figure 2:**
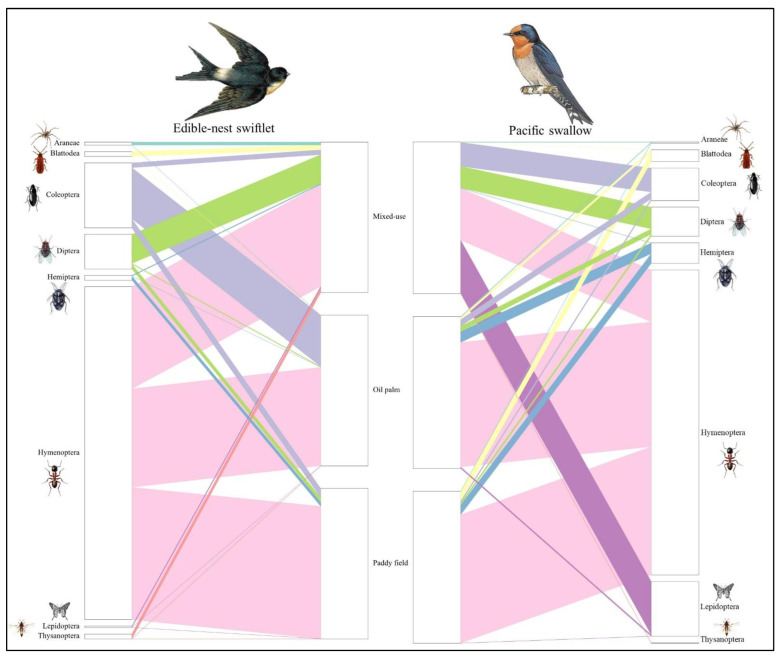
Bipartite diagram of dietary variation between habitat landscapes of two species of aerial insectivorous birds in Peninsular Malaysia.

The diets of both aerial insectivorous bird species across the three distinct habitats were primarily composed of arthropod prey from class Insecta comprising 13 orders, including Blattodea (cockroaches), Coleoptera (beetles), Dermaptera (earwigs), Diptera (flies), Ephemeroptera (mayflies), Hemiptera (true bugs), Hymenoptera (wasps, bees, ants), Lepidoptera (moths, butterflies), Odonata (dragonflies, damselflies), Orthoptera (grasshoppers, crickets), Psocodea (booklice, barklice), Thysanoptera (thrips), and Trichoptera (caddisflies). Additionally, prey from class Arachnida was identified with five orders including Araneae (spiders), Ixodida (ticks), Mesostigmata (mites), Oribatida (beetle mites) and Trombidiformes (spider mites), along with class Chilopoda represented by the order Scolopendromorpha (centipedes). Of the 19 total orders identified across the three habitats, six were dominant, each contributing over 5% to the overall diets of aerial insectivorous birds. These dominant orders included Coleoptera (beetles), Diptera (flies), Blattodea (cockroaches, termites, and mantids), Hemiptera (true bugs), Hymenoptera (ants, bees, and wasps), and Lepidoptera (butterflies and moths). Hymenoptera made up the largest proportion among orders and were consumed in the diet of aerial insectivorous birds, with varying consumption between the regions (Figure 2).

### 3.2. Variation in Prey Composition and Diversity Across Habitats Consumed by Aerial Insectivores

The composition of prey taxa varied significantly between the three habitats. The mixed-use habitat exhibited the highest diversity at broader taxonomic levels with three classes, 18 orders, 168 families, 180 genera, and 101 insect species. Paddy fields demonstrated moderate diversity, with two classes, 13 orders, 119 families, 106 genera, and 100 species identified. In contrast, oil palm plantations exhibited the lowest diversity, with two classes, 11 orders, 82 families, 66 genera, and 94 species. Two-way PERMANOVA analysis of the dietary composition by order rank across all three habitats showed support of significant differences in prey composition between the two bird species (F = 2.078, R^2^ = 0.528, df = 2, *p* = 0.0232), emphasising the influence of habitat type on dietary composition.

**Figure 3:**
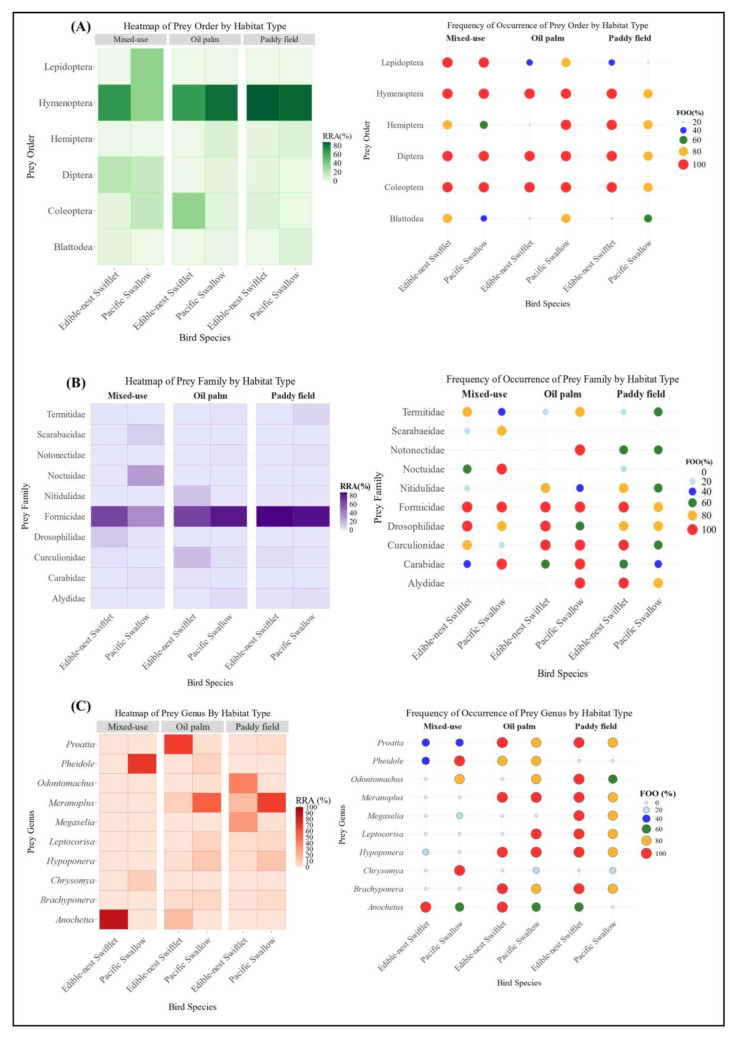
(**A**) Prey composition (main order level); (**B**) Prey composition (main family level); (**C**) Prey composition (main genus level) of two species of aerial insectivorous birds between habitats.

Mixed-use habitats had the highest prey diversity, with Hymenoptera as the dominant prey, making up 67.71% of the Edible-nest swiftlet’s diet and 34.25% of the Pacific swallow’s diet. Both species had a frequency of occurrence (FOO) of 100% for Hymenoptera, indicating its presence in every sampled individual (Figure 3A). Diptera was another prominent prey order, contributing 19.26% and 14.29% to the diets of Edible-nest swiftlets and Pacific swallows, respectively (FOO = 100%). Lepidoptera played a significant role in the Pacific swallow’s diet, comprising 34.98%, but its presence was minimal in other habitats. Thysanoptera also showed higher proportions in mixed-use habitats, contributing 3.12% to the Edible-nest swiftlet’s diet and 0.23% to the Pacific swallow’s diet, whereas it was nearly absent in oil palm and paddy fields. Additionally, certain prey orders, such as Scolopendromorpha and Mesostigmata, were found exclusively in mixed-use habitats.

Hymenoptera was the most abundant prey order in paddy fields, contributing 87.98% of the Edible-nest swiftlet’s diet (FOO = 100%) and 84.44% of the Pacific swallow’s diet (FOO = 80%). Blattodea was also a notable prey order in paddy fields, making up 6.20% of the Pacific swallow’s diet, while its contribution to the Edible-nest swiftlet’s diet was minimal. Coleoptera and Hemiptera were also significant in this habitat, with both species consuming them (FOO = 100%). The Edible-nest swiftlet consumed more Coleoptera (6.08%) and Hemiptera (2.62%), whereas the Pacific swallow showed a stronger preference for Hemiptera (6.37%) and a lower proportion of Coleoptera (2.62%) (Figure 3A).

In oil palm habitats, Hymenoptera remained a dominant dietary component, making up 81.96% of the Pacific swallow’s diet and 65.36% of the Edible-nest swiftlet’s diet (FOO = 100%) (Figure 3A). Coleoptera was the second most consumed order, contributing 33.92% to the Edible-nest swiftlet’s diet and 4.41% to the Pacific swallow’s diet (FOO = 100%). In contrast, mixed-use habitats had lower proportions of Coleoptera, with 15.76% in the Pacific swallow’s diet and 3.22% in the Edible-nest swiftlet’s diet (FOO = 100%). Hemiptera was also present in oil palm plantations, comprising 7.03% of the Pacific swallow’s diet and a negligible 0.002% for the Edible-nest swiftlet. In mixed-use habitats, Hemiptera contributed less than 1% for both species, highlighting distinct prey preferences across different environments.

Formicidae was the most consumed family in each habitat in both bird species. However, statistical analysis conducted at the family level using a two-way PERMANOVA (F = 1.7564, R^2^ = 0.5014, df = 2, *p* = 0.0436) showed significant differences in diet composition between the three habitats. These data indicate that habitat type significantly influences dietary patterns at the order and family levels, reflecting distinct habitat-dependent prey preferences. The dietary overlap at the family level of arthropod preys consumed between the mixed-use habitat and the oil palm plantation was the lowest (Ojk = 0.06). Meanwhile, the dietary overlap at the family level between two other habitat pairs: mixed-use habitat and paddy fields, and paddy fields and oil palm plantations, each recorded value of (Ojk = 0.09). Thus, the Pianka Index, consistent with the RRA data, indicates that dietary overlap is higher between monoculture habitats (e.g., oil palm plantations and paddy fields), though at a very minimal extent. Conversely, dietary overlap is lowest between habitats with distinct levels of diversity, such as mixed-use urban–rural landscapes compared to monoculture plantation habitats.

In the mixed-use habitat of Bentong, Formicidae was the dominant prey family for both aerial insectivores, comprising 65.09% of the Edible-nest swiftlet’s diet and 35.74% of the Pacific swallow’s (both 100% FOO) (Figure 3B). Other significant families included Noctuidae and Scarabaeidae in the Pacific swallow’s diet and Drosophilidae in the Edible-nest swiftlet’s diet. The habitat had the highest genus diversity, as shown by the Shannon Index (H’ = 1.827), reflecting a rich dietary community. *Anochetus* sp. dominated the Edible-nest swiftlet’s diet (86.65%), contributing to a low evenness index (E = 0.035), though other genera supported the habitat’s richness. The Pacific swallow’s diet was more varied, including higher proportions of *Pheidole* sp., *Chrysomya* sp., *Isomyia* sp., *Scieropepla* sp., and *Camponotus* sp., highlighting diverse prey selection in this habitat. In the paddy field habitat, Formicidae dominated the diets of both bird species, making up 87.80% of the Edible-nest swiftlet’s diet (FOO = 100%) and 84.25% of the Pacific swallow’s diet (FOO = 80%) (Figure 3B). The habitat showed moderate genus diversity, Shannon Index (H’ = 1.697) and higher evenness (E = 0.0515) compared to the mixed-use habitat, with the lowest genus dominance (D = 0.252), indicating a balanced prey distribution. Other significant genera included *Meranoplus* sp., *Odontomachus* sp., and *Hypoponera* sp., which also contributed notably to the diets of both species. The prominence of Formicidae highlights its crucial role in the prey composition within the paddy field ecosystem. In the oil palm habitat of Kulai, the diets of both bird species were dominated by Formicidae, making up 81.81% of the Pacific swallow’s diet and 65.32% of the Edible-nest swiftlet’s diet, both with FOO = 100%. Curculionidae and Nitidulidae were also significant in the Edible-nest swiftlet’s diet, while the dominance of Formicidae was driven by *Proatta* sp. and *Anochetus* sp. in this species. The Pacific swallow’s diet was primarily composed of *Meranoplus* sp. and *Hypoponera* sp., with additional contributions from *Leptocorisa* sp., and *Musca* sp. Meanwhile, *Polytus* sp., *Elaeidobius* sp. and *Anoplolepis* sp. were part of the diets for both species. The oil palm habitat showed the lowest genus diversity, Shannon Index (H’ = 1.38), highest dominance (D = 0.401), and a moderate evenness index (E = 0.0602), indicating limited diversity but a slightly balanced distribution among the genera.

### 3.3. Dominance of Specific Insect Species in Aerial Insectivore Diets Across Habitats

At the species level, both insectivorous bird species demonstrated distinct prey preferences across the three habitats. In the mixed-use urban–rural habitat, a total of 101 insect species were identified in their diets, representing the highest diversity among the habitats. In the paddy field habitat, 100 insect species were recorded, while the oil palm habitat supported the lowest diversity, with 94 insect species consumed.

**Figure 4:**
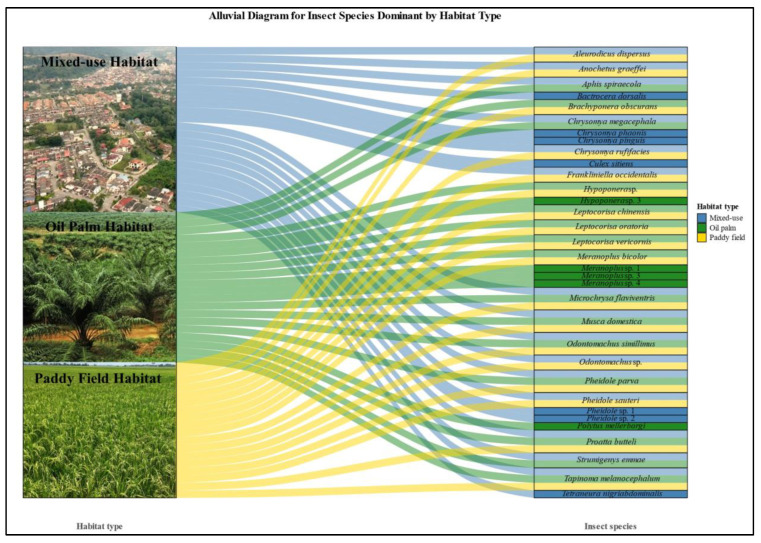
Alluvial diagram to demonstrate the interaction of specific dominance insect species and overlap dominance insect species detected across three different habitats.

The mixed-use habitat showed the highest insect species diversity and dietary niche breadth, as indicated by the highest Levins’ Index value (B = 6.13), reflecting a wide range of prey species consumed by the insectivorous birds. For Edible-nest swiftlets, the dominant prey species included *Anochetus graeffei*, *Proatta butteli*, and *Microchrysa flaviventris*. Unique species consumed only in this habitat were *Culex sitiens* and *Tetraneura nigriabdominalis*. For Pacific swallows, key prey species were *Pheidole* sp. 1, *Pheidole* sp. 2, *Chrysomya megacephala*, *Bactrocera dorsalis*, *Chrysomya pinguis*, and *Chrysomya phaonis*, though *Chrysomya megacephala* was not exclusive to this habitat (Figure 4).

The paddy field habitat showed a moderate dietary niche breadth (Levins’ Index B = 5.17), indicating intermediate prey diversity. *Meranoplus bicolor* was the dominant prey for both species, with *Pheidole sauteri*, *Odontomachus simillimus*, *Pheidole parva*, and *Odontomachus* sp. being key prey for the Edible-nest swiftlet. For the Pacific swallow, additional important species included *Hypoponera* sp., *Proatta butteli*, *Leptocorisa oratoria*, and *Leptocorisa chinensis* (Figure 4). While the paddy field habitat supported moderate prey diversity, no uniquely dominant insect species were observed, as all major prey were also present in other habitats. 

In contrast, the oil palm habitat had the narrowest dietary niche breadth (Levins’ Index B = 1.75), driven by the dominance of specific prey. *Proatta butteli* and *Meranoplus bicolor* formed the majority of the diet for both bird species in this habitat. Five insect species, including *Hypoponera* sp. 3, *Meranoplus* sp. 1, *Meranoplus* sp. 3, *Meranoplus* sp. 4, and *Polytus mellerborgi*, were unique and dominant in the Kulai region, significantly contributing to the diets of both bird species (Figure 4). *Hypoponera* sp. 3 and *Polytus mellerborgi* were key components of the diet for both Edible-nest swiftlets and Pacific swallows. The *Meranoplus* species collectively formed a substantial part of the diet, with notable contributions from *Meranoplus sp*. 1, *Meranoplus* sp. 3, and *Meranoplus* sp. 4. These findings emphasize the ecological distinctiveness of the Kulai region, where specific insect species dominate and serve as crucial dietary resources for aerial insectivorous birds.

Five insect species, belonging to order Diptera (*Microchrysa flaviventris* and *Musca domestica*) and order Hymenoptera *(Odontomachus simillimus*, *Pheidole parva*, and *Proatta butteli*), overlapped across all three habitat types. These species exhibited broad ecological tolerance, thriving in mixed-use habitats, oil palm plantations, and paddy fields, though their abundance varied across habitats. This overlap highlights the adaptability of these species and their potential roles in ecosystem processes such as nutrient cycling, pest control, and soil structuring, underscoring their ecological importance in agricultural and mixed-use landscapes. Several insect species overlapped between mixed-use habitats and paddy fields, while absent within oil palm plantations. Species such as *Aleurodicus dispersus*, *Anochetus graeffei*, *Chrysomya rufifacies*, *Frankliniella occidentalis*, *Odontomachus* sp., and *Pheidole sauteri* were detected in both mixed-use and paddy fields, though their relative abundances varied. For mixed-use and oil palm habitats, overlapping species included *Aphis spiraecola*, *Strumigenys emmae*, and *Chrysomya megacephala*. Overall, mixed-use habitats supported the most overlapping species, while paddy fields had fewer shared species, and oil palm plantations exhibited minimal overlaps, reflecting differences in habitat conditions and suitability. Species overlapping between monoculture habitats (oil palm plantations and paddy fields), demonstrated notable adaptations to these environments while being absent in mixed-use habitats. Key overlapping species included *Meranoplus bicolor*, *Leptocorisa chinensis*, *Leptocorisa oratoria*, *Brachyponera obscurans*, *Leptocorisa vericornis*, *Hypoponera* sp., and *Tapinoma melanocephalum*. These species showed varying degrees of abundance across the two monoculture habitats, reflecting their ecological specialization within simplified and resource-specific environments. While *Meranoplus bicolor* and *Leptocorisa* species were prominent in both habitats, *Brachyponera obscurans* and *Hypoponera* sp. showed stronger associations with either paddy fields or oil palm plantations. *Tapinoma melanocephalum* was unique to monocultures, being present in both habitats but primarily associated with paddy fields. These findings highlight the adaptations of these species to monoculture systems while highlighting their absence in the more diverse mixed-use landscapes.

## 4. Discussion

The diets of two species of aerial insectivorous birds across three different types of habitats were significantly different in the diversity of arthropod prey within the diet of each bird and the proportion of major arthropod prey species within the diets. Both bird species mostly fed on the ant family, order Hymenoptera, in all three habitats. The dominance by ants was most similar between monocultural landscapes, such as oil palm plantations and paddy fields. The differences in the dietary preferences of the two species of aerial insectivores across mixed-use landscapes, paddy fields, and oil palm plantations were likely driven by differences in the prey availability, habitat structures and ecological functions that support each of the ecosystems. Each of these three habitats provides distinct taxa as available prey for aerial insectivores, which mixed-use habitats provide the most diverse prey options as a food resource to the bird species with up to 18 different orders. Paddy field habitats provide moderate diversity of prey, and the ecosystems support several arthropod taxa and offer the most abundance of certain taxa such as Blattodea, Coleoptera, Diptera, Hemiptera and Hymenoptera. In contrast, the oil palm plantations offer the least diversity of dietary prey options, and taxa within the ecosystem were limited to a small number of arthropod species and the highly dominant Hymenoptera order. These three landscapes are likely to have distinct ecological characteristics, which makes their biodiversity distinct from each other, such as differences in abiotic (e.g., vegetation structures, and climatic conditions) and biotic (competitors, availability of food resources and reproduction sites) factors, which together affect the accessibility of specific insect taxa [39].

Mixed-use landscapes that integrate natural forests, agricultural zones, and human infrastructure sustain a higher insect diversity compared to monocultures or heavily urbanised environments. Mixed-use landscapes create a mosaic of environmental conditions that alleviate the ecological pressures associated with land-use change, contributing to the stabilisation of insect populations [40]. The mixed-use habitat in Bentong is dominated by forested areas (72%), with the contribution of other green spaces (oil palm plantation and agriculture crops) and urban settlements. By incorporating of high proportion of both natural and cultivated areas, these create a heterogeneous habitat provide essential refuges for diverse arthropod taxa, safeguarding them from declines commonly observed in more homogeneous ecosystems. For example, the region of Bentong may possess a higher diversity of insect orders in the mixed-use ecosystem, as indicated by the greater variety of taxa ingested by both species of birds, especially the orders Coleoptera, Blattodea, Diptera, Lepidoptera, and Thysanopteras. These conditions shape the species-specific adaptation of aerial insectivorous birds like Edible-nest swiftlets and Pacific swallows, enabling them to exploit the diverse prey base availability in mixed-use habitat. 

Monoculture habitats, such as paddy fields and oil palm plantation have significantly lower arthropod diversity compared to mixed-use habitat due to the simplification of habitat structure and homogenisation of resources. The simplified vegetation structure in monoculture systems will decrease the habitat complexity by reducing the arthropod species richness and promoting the dominance of a few species [41]. For instance, homogenous paddy fields mixed with large areas of urbanisation-dominated landscapes in Seberang Perai may reduce the microhabitat or niche of insect taxa. Agricultural landscapes adjacent to residential developments experience profound ecological shifts, resulting in significant impacts on insect populations. Urbanisation near these zones typically drives habitat fragmentation and biodiversity loss as human activities reshape the environment, rendering it less suitable for sustaining diverse insect communities [42]. A major consequence of urban encroachment is the disruption of insect movement and resource availability. For example, flying insects, including pollinators, predators, and parasitoids, encounter physical barriers such as roads, buildings, and fragmented habitats, restricting their access to food and nesting sites. Research conducted in Rome demonstrated that urbanisation heightened spatial barriers, leading to declines in the abundance and diversity of essential predator and parasitoid groups [43]. These ecosystems promote the dominance of arthropod taxa that have broad dietary and habitat preferences, which will drive functional homogenisation, reducing diversity within insect communities [44]. The dominance of certain Hymenoptera species, such as *Meranoplus bicolor*, *Pheidole sauteri*, *Odontomachus simillimus*, *Pheidole parva*, *Hypoponera* sp., and *Odontomachus* sp., in the diet of aerial insectivores in paddy field demonstrates the influence of monoculture habitats in promoting certain arthropod taxa that adapt well to homogeneous ecosystems. Consequently, the resilience of these communities to environmental changes is compromised, diminishing the essential ecosystem services, such as pollination and pest control, that support agricultural productivity. The lack of dominance by specific insect species in paddy fields can be attributed to the seasonal nature of rice cultivation, which creates periodic fluctuation in arthropod resource availability. The prey availability within paddy fields fluctuates with the phase of paddy, including pre-planting, flowering and harvesting that typically allows for two planting seasons per year. Meanwhile, oil palm plantations in Kulai are primarily defined by their monoculture vegetation structure that dominates the landscapes. However, the presence of limited forest patches may contribute slightly to the creation of microhabitats. These environments provide essential shelter, nesting sites, and food resources for particularly beetles, thereby contributing to the highest diversity of beetles in the diet of aerial insectivorous birds observed within oil palm ecosystems in Kulai. Coleopteran beetles serve various ecological roles as herbivores, decomposers, and pollinators. Within oil palm plantation ecosystems, decomposer species utilise abundant decaying organic matter, while herbivorous beetles, such as those from the family Curculionidae and Erirhinidae, feed on roots and decaying plant materials [45].

The consumption of Hymenoptera by both aerial bird species was prominent in the oil palm and paddy field habitats, as well as by the Edible-nest swiftlet in the mixed-use habitat. Key species included *Pheidole* sp., *Meranoplus bicolor*, *Proatta butteli*, *Hypoponera* sp., and *Odontomachus simillimus*, collectively making up nearly two-thirds of the total diet. This suggests that these habitats attract ants and flying ants [46,47]. In agricultural areas, ants play a crucial role in seed dispersal for certain plantations by collecting seeds from plants and transporting them to new locations. This process supports plant colonisation and spread, contributing to the maintenance of botanical diversity and aiding ecosystem restoration in agriculture [47,48]. The order Hymenoptera encompasses ants, bees and wasps, which have a perennial presence and generalist behaviours [49]. Therefore, Hymenopterans frequently contribute the most insects consumed by aerial insectivorous birds as they are found in urban, forest and agricultural ecosystems [50] where their proliferation is facilitated by the variety of vegetation and year-round resource availability.

Edible-nest swiftlets are aerial foragers that predominantly consume flying insects during flight. They typically soar at elevated altitudes, potentially restricting their diet to insects that are more prevalent at these heights, such as Hymenoptera (bees, wasps, ants), which are highly mobile and plentiful in the upper strata of vegetation and wide altitude within the flight boundary layers in air space. Within the order Hymenoptera, Formicidae is one of the families that have a higher altitude of flight, ranging from 2 m up to 43 m [51]. This could be the main explanation for the dominancy of Formicidae among the diets of aerial insectivorous birds. In contrast, Pacific swallows exhibit greater agility and versatility as generalist foragers, and are adept at capturing insects in proximity to the ground, near aquatic environments, or within foliage. Their capacity to forage across diverse microhabitats may elucidate the greater variability in their diet, encompassing orders rather than Hymenoptera such as Lepidoptera (moths and butterflies), Diptera (flies), and Hemiptera (true bugs), which are typically located at lower elevations or in proximity to aquatic environments [52,53,54].

A recently developed COI primer set, LCOI-1490/COI-CFMRa, known as ANML, has emerged as a powerful tool for DNA metabarcoding in dietary studies, particularly in insectivorous species. The ANML primers target a ~180 bp region of the COI gene and significantly enhance the detection of arthropod DNA, offering high taxonomic resolution down to the species level. This precision allows for more reliable and comprehensive identification of prey, capturing a broader range of taxa, including rare and low-abundance species, which are often overlooked by other primers. Using high-throughput sequencing with the ANML primer, this study identified over 1833 unique ASVs, spanning 245 species across 230 families. Such detailed detection is invaluable for understanding the dietary patterns of aerial insectivorous birds and their ecological interactions. Compared to previous studies, our findings provide a more detailed understanding of dietary composition by identifying key agricultural pests such as *Leptocorisa chinensis* and *Polytus mellborgi*, highlighting the role of aerial insectivores in pest control. In contrast, ref. [24] identified the diet of Edible-nest swiftlets only at the order level, and refs. [25,55] reported generalist insect consumption by Pacific swallows without distinguishing pest species. Our use of high-throughput sequencing revealed habitat-specific dietary patterns, with mixed-use landscapes supporting greater prey diversity compared to more specialised diets in monoculture systems. This approach also detected temporal shifts in dietary composition linked to agricultural cycles, a pattern not observed in previous studies. These findings demonstrate the ecological flexibility and adaptive foraging strategies of aerial insectivorous birds, providing species-level dietary insights that enhance our understanding of their role in ecosystem services and agricultural sustainability.

The higher consumption of *Culex sitiens* as a specific dominant species in mixed-use habitats, along with key agricultural pests such as *Polytus mellborgi*, *Leptocorisa chinensis*, *Leptocorisa oratoria*, *Leptocorisa vericornis*, *Leucinodes orbonalis*, and *Tetraneura nigriabdominalis*, highlights the critical role of aerial insectivorous birds in controlling pest populations. This activity not only supports healthier agricultural ecosystems but also aids in reducing the spread of vector-borne diseases, thereby contributing to improved public health and environmental stability. 

Understanding the diet of aerial insectivorous birds in agricultural ecosystems is critical, as these birds can act as natural biological control agents by consuming pest species that negatively affect crop yields. By preying on a wide range of arthropods, including agricultural pests, they contribute to ecosystem stability and reduce the need for synthetic pest control methods. The comprehensive taxonomic resolution offered by ANML primers supports ecological research by uncovering fine-scale dietary patterns, informing conservation efforts, and highlighting the role of these birds in promoting agricultural sustainability. 

## 5. Conclusions

Aerial insectivorous birds, such as Edible-nest swiftlets and Pacific swallows are vital components of various ecosystems, including agriculture landscapes. These birds contribute significantly to ecosystem stability by consuming a wide range of flying arthropods, naturally regulating insect populations and promoting a balance biodiversity. Their ability to adapt to diverse types of habitats, from mixed-use landscapes to oil palm plantations and paddy fields, emphasises their ecological importance in maintaining ecosystem functions in wide landscape types. In agricultural settings, they play a key role in enhancing crop productivity by controlling pests, offering a sustainable and natural alternative to intensive management practices. Protecting aerial insectivorous birds through habitat conservation and fostering biodiversity is essential to maintaining their ecological contributions and supporting the health and resilience of agricultural and natural ecosystems.

## Data Availability

The data that support the findings of this study are available on request from the corresponding author.

## Supplementary Materials

The following supporting information can be downloaded at https://www.mdpi.com/article/10.3390/ani15070974/s1, Supplementary File S1: metrics to quantify the diet taxa abundance; Supplementary File S2: Table of the details of the dietary preferences by species of aerial insectivorous birds across three habitats.

## Abbreviations

The following abbreviations are used in this manuscript:

DNA	Deoxyribonucleic acid
COI	Cytochrome c oxidase subunit 1
ASV	Amplicon sequence variants
FOO	Frequency of occurrence
RRA	Relative read abundance
HTS	High Throughput Sequencing
