# Correction: Arazmi et al. DNA Metabarcoding Unveils Habitat-Linked Dietary Variation in Aerial Insectivorous Birds. *Animals* 2025, *15*, 974

**DOI:** 10.3390/ani15162383

**Published:** 2025-08-14

**Authors:** Fatihah Najihah Arazmi, Nor Adibah Ismail, Ummi Nur Syafiqah Daud, Mohammad Saiful Mansor

**Affiliations:** 1 Department of Biological Sciences and Biotechnology, Faculty of Science and Technology, Universiti Kebangsaan Malaysia, Bangi 43600 UKM, Selangor, Malaysia; n.fatihahnajihah@gmail.com; 2 Department of Earth Sciences and Environment, Faculty of Science and Technology, Universiti Kebangsaan Malaysia, Bangi 43600 UKM, Selangor, Malaysia; nadibahismail@gmail.com (N.A.I.); ummisyafiqah96@gmail.com (U.N.S.D.)

## Text Correction

There was an error in the original publication [1]. The sequencing read length was incorrectly stated to be 2 × 150 bp instead of the correct 2 × 250 bp.

A correction has been made to Section 2.3. DNA Extraction, Amplification and Sequencing, Paragraph 1.

Corrected Paragraph:

“Sequencing was conducted on an Illumina MiSeq platform (2 × 250 bp, Illumina, San Diego, CA, USA) following standard protocols.”

There was an error in the original publication [1]. The reference [28] was incorrectly written as Mansor, Halim, Abdullah and Ramli [28]. It was thus incorrectly written with all author surnames instead of et al.

A correction has been made to Section 2.4. Bioinformatics and Data Analysis, Paragraph 2.

Corrected Paragraph:

“Sequences showing a minimum 98% similarity were classified at the species level, those with 95% similarity at the genus level, and sequences with 90% similarity at the family level, following the methods of Mansor et al. [28].”

The authors state that the scientific conclusions are unaffected. This correction was approved by the Academic Editor. The original publication has also been updated.
