# Supplementary material for: DNA Metabarcoding Unveils Habitat-Linked Dietary Variation in Aerial Insectivorous Birds
Source: Animals (Basel). 2025 Mar 27;15(7):974. doi: 10.3390/ani15070974 (PMC11987892; doi:10.3390/ani15070974)
Supplement: Supplementary file 1 [file animals-15-00974-s001.zip › animals-3457764-supplementary.pdf]

Suplementary File S1: metrics to quantify the diet taxa abundance

1. **Formula for frequency of occurrence (FOO)**

The calculation of frequence of occurrence (%FOO) was involved with the number of samples that contain in a given diet taxa over the sum of detections of a particular prey item for a bird species by the total number of individuals of that bird species included in the study (*N* = 15). The following equation is used to calculate %FOO for a diet taxon:

Where *N* is the number of samples, *n* is the occurrence function such that *ni,k* = 1 if food item *i* occurs in sample *k*, and 0 if not.

$$\% FOO = \frac{n_{i,k}}{N} \times 100\%$$

2. **Formula for relative read abundance (RRA)**

The sequence count data are used to calculate the relative abundance of each diet taxa, i.e. the percentage of taxa detected in the diet belonging to each species of aerial insectivorous birds. Where N is the number of samples, T is the number of food items, ni,k is the number of sequences of food item i in sample k.

$$\% RRA_i = \sum_{k=1}^{N} \frac{n_{i,k}}{\sum_{i=1}^{T} n_{i,k}} \times 100\%$$

Supplementary File S2: Table of the details of the dietary preferences by species of aerial insectivorous birds across three habitats.

Habitat	Mixed-use	Mixed-use	Paddy field	Paddy field	Oil palm	Oil palm
Bird Species	Edible-nest swiftlet	Pacific swallow	Edible-nest swiftlet	Pacific swallow	Edible-nest swiftlet	Pacific swallow
Prey taxa	RRA (%)	RRA (%)	RRA (%)	RRA (%)	RRA (%)	RRA (%)
Order: Araneae						
Family: Ctenidae						
Amauropelma Krabi	0.10					
Family: Pholcidae						
Modisimus sp.	0.49					
Family: Sparassidae						
Pandercetes malleator		0.04				
Order: Ixodida						
Family: Ixodidae						
Rhipicephalus sanguineus	0.17			0.01		
Order: Trombidiformes						
Family: Cheyletidae						
Cheyletus malaccensis			0.03			

Family: Tetranychidae

Oligonychus sp.			0.0002		
Order: Scolopendromorpha					
Family: Scolopendridae					
Scolopendra subcrustalis		0.07			
Class: Insecta					
Order: Blattodea					
Family: Blaberidae					
Opisthoplatia orientalis		0.01			
Opisthoplatia sp.		0.0004			
Family: Rhinotermitidae					
Schedorhinotermes sp.	0.38				
Family: Termitidae					
Dicuspiditermes sp.			0.0002	0.68	
Macrotermes jeanneli					0.004
Macrotermes malaccensis	0.01	0.01			0.004
Macrotermes sp. 1	0.01	0.01			0.004
Macrotermes sp. 2	0.01	0.01			0.004
Macrotermes sp. 3	0.01	0.01			
Macrotermes sp. 4	0.01	0.01			
Microcerotermes sp.			0.0003	1.25	

Pericapritermes sp.	0.68	0.003				
Pericapritermes sp. 1						0.02
Pericapritermes sp. 2						0.003
Pericapritermes sp. 3						0.13
Order: Coleoptera						
Family: Apionidae						
Kalcapion sp.			0.003			
Family: Cerambycidae						
Rhagium sp.		0.08				
Family: Curculionidae						
Elaeidobius kamerunicus					0.98	0.02
Elaeidobius sp.				0.03	1.68	0.24
Euplatypus parallelus			0.093			
Euplatypus sp.			0.047			
Euwallacea sp.			0.001			
Fania sp.				0.02	0.002	
Polytus mellerborgi						1.465
Polytus sp.						1.34
Polytus sp. 1						0.002
Polytus sp. 2						0.002
Polytus sp. 3						0.208
Xyleborus perforans			0.008			

Family: Histeridae					
Saprinus sp.		0.0005			
Family: Hydrophilidae					
Cercyon laminatus		0.003			
Cheyletus sp.		0.02			
Family: Nitidulidae					
Carpophilus mutilatus		0.02			0.05
Carpophilus sp.					0.05
Carpophilus obsoletus		0.026	0.02	0.02	
Epuraea luteola		0.054			
Epuraea ocularis					
Family: Staphylinidae					
Atheta parvipennis		0.0002			
Atheta sp.		0.0001			
Erymus gracilis		0.001			
Erymus sp.		0.0001			
Order: Dermaptera					
Family: Labiduridae					
Labidura riparia	0.17		0.01		
Labidura sp.	0.03		0.005		
Order: Diptera					
Family: Agromyzidae					

Family: Calliphoridae						
Chrysomya bezziana		0.36				
Chrysomya chani		1.18				
Chrysomya megacephala		23.29				0.18
Chrysomya phaonis		4.33				
Chrysomya pinguis		1.54				
Chrysomya rufifacies		2.03		0.27		0.112
Chrysomya sp.		8.88		0.25	0.003	
Hemigymnochaeta sp.				0.02		
Sarconesiopsis sp.				0.02	0.002	
Tricyclea sp.				0.02		
Verticia orientalis				0.02	0.002	
Verticia sp.				0.02	0.002	
Family: Cecidomyiidae						
Mayetiola hordei	0.08					
Family: Ceratopogonidae						
Culicoides actoni	0.41					
Culicoides orientalis	0.07					
Culicoides sp.	0.08					
Family: Chironomidae						
Chironomus flaviplumus				0.11		

Chironomus javanus			0.05		
Chironomus kiiensis			0.02		
Chironomus striatipennis			0.05		
Chironomus vitellinus			0.01		
Chironomus sp.			0.12	0.1	
Kiefferulus calligaster			0.004		
Kiefferulus glauciventris			0.001		
Kiefferulus sp.			0.01		
Kiefferulus tainanus			0.02		
Parachironomus sp.		0.03			
Parakiefferiella sp.	0.004				
Procladius sp.			0.01		
Synorthocladius sp.	0.004				
Tanytarsus oscillans					0.01
Thienemanniella sp.	0.07				
Family: Chloropidae					
Aphanotrigonum nigripes			0.03		
Eutropha sp.	0.02				
Hippelates sp.		0.183			
Thaumatomyia sp.			0.002		
Tricimba sp.			0.03		
Family: Culicidae					
Aedes albopictus		0.66			
Aedes sp.		0.687			

Anopheles aconitus			0.0004	
Anopheles albitarsis			0.001	
Anopheles barbirostris			0.003	
Anopheles campestris			0.01	
Anopheles gambiae				0.02
Anopheles sp.	0.029	0.036	0.463	0.106
Anopheles vagus			0.01	
Anopheles wejchoochotei			0.003	
Armigeres sp.		0.0334		
Armigeres sp. 1		0.14		
Armigeres sp. 2		0.14		
Armigeres subalbatus		0.95		
Culex gelidus			0.03	
Culex Lophoceraomyia			0.002	
Culex pseudovishnui	3.61		0.003	
Culex sitiens				
Culex sp.	0.538		0.095	
Culex tritaeniorhynchus			0.005	
Culex vishnui			0.09	
Mansonia bonneae			0.002	
Mansonia sp.			0.009	
Mansonia uniformis			0.01	
Family: Dolichopodidae				
Chrysotus sp.	0.312			

Family: Drosophilidae						
Drosophila latifshahi					0.0001	
Drosophila polychaeta					0.0001	
Drosophila sp.	0.463	0.036	0.037		0.002	0.006
Phortica sp.	0.176	0.214				
Rhinoleucophenga sp.	0.029	0.036				
Stegana sp.	0.055					
Zygothrica sp.					0.0001	
Family: Ephydridae						
Lamproscatella sp.			0.002			
Family: Fanniidae						
Filinota sp.				0.017		
Family: Heleomyzidae						
Heteromyza sp.		0.03				
Family: Hybotidae						
Bicellaria sp.					0.008	
Family: Keroplatidae						
Macrocera sp.		0.07				
Family: Limoniidae						
Neolimnophila sp.	0.83	0.004	0.06	0.024	0.024	
Family: Muscidae						

Atherigona sp.		0.33			0.042	0.002
Azelia sp.						
Coenosia sp.			0.001			
Haematobia sp.				0.021		
Hydrotaea sp.			0.003			
Musca domestica		0.82		0.19		2.35
Polietina sp.		0.06				
Spilogona sp.	0.012					
Synthesiomyia nudiseta		0.17				
Synthesiomyia sp.		0.055	0.008			
Thricops sp.						
Family: Mycetophilidae						
Clastobasis sp.		0.009				
Family: Phoridae						
Diplonevra sp.		0.147				
Megaselia sp.		0.247	30.23	2.09		
Family: Psychodidae						
Microvelia sp.			0.13			
Sycorax sp.	0.031					
Family: Rhiniidae						
Borbororhinia bivittate		0.68				
Borbororhinia sp.		0.18				
Isomyia sp.	0.019	2.71				
Strongyloneura sp.		0.061				

Family: Sarcophagidae						
Sarcophaga sp.		0.54				
Family: Sepsidae						
Dicranosepsis sauteri					0.01	
Dicranosepsis sp.	0.36				0.065	
Family: Stratiomyidae						
Hermetia illucens						0.09
Hermetia sp.						0.323
Microchrysa flaviventris	3.06		0.04		0.02	
Microchrysa sp.						
Micropygomyia sp.	0.46		0.041		0.013	
Family: Tachinidae						
Family: Tephritidae						
Bactrocera carambolae		0.1				
Bactrocera dorsalis		1.43				
Bactrocera minax			0.03	0.02	0.02	
Bactrocera sp.		0.41	0.02	0.02		
Zeugodacus cucurbitae		0.41				
Zeugodacus sp.		0.11				
Order: Hemiptera						
Family: Aleyrodidae						
Aleurodicus dispersus	1.05		0.1			

Aleurodicus floccissimus			0.001	0.001		
Aleurodicus rugioperculatus	0.21					
Aleurodicus sp.	0.91		0.07			
Aleurotrachelus atratus	0.09					
Family: Alydidae						
Leptocorisa chinensis			1.74	2.17		3.34
Leptocorisa oratoria			2.39	2.99		4.54
Leptocorisa sp.			3.3	4.95		7.08
Leptocorisa vericornis			0.22	0.27		0.42
Family: Aphididae						
Aphis spiraecola	1.11				0.03	
Aphis sp.					0.03	
Hysteroneura setariae	0.68					0.05
Hysteroneura sp.	0.1					
Pterocallis sp.	0.02					
Tetraneura nigriabdominalis	2.25	0.01				
Tetraneura sp. 1	0.25					
Tetraneura sp. 2	0.25					
Tetraneura sp. 3	0.25					
Tetraneura sp. 4	0.25					
Tetraneura sp. 5	0.25					
Tetraneura sp. 6	0.25					
Tetraneura sp. 7	0.25					

Family: Carsidaridae					
Carsidara limbate			0.0002		
Carsidara sp.			0.0001		
Family: Cicadellidae					
Amrasca sp.		0.02			
Amrasca splendens			0.003		
Cicadella sp.		0.02			
Cicadella viridis		0.07			
Hishimonus phycitis	0.21				
Hishimonus sp.	0.03				
Family: Delphacidae					
Sogatella furcifera			0.002		
Sogatella sp.			0.002		
Family: Miridae					
Cyrtorhinus lividipennis					0.004
Cyrtorhinus sp.					0.003
Family: Notonectidae					
Anisops sp.			0.04	0.3	
Family: Pentatomidae					
Rhaphigaster nebulosa	0.08				
Family: Plataspidae					

Brachyplatys subaeneus						0.03
Brachyplatys sp.						0.02
Family: Pyrrhocoridae						
Ectatops rubiaceus		0.05				
Family: Veliidae						
Microvelia douglasi			0.18			
Order: Hymenoptera						
Family: Agaonidae						
Kradibia sp.						
Family: Apidae						
Amegilla calceifera			0.001			
Amegilla thorogoodi			0.00			
Amegilla zonata			0.00			
Apis cerana	0.21		0.02		0.004	
Geniotrigona sp.		0.02				
Geniotrigona thoracica		0.04				
Family: Braconidae						
Apanteles sp.			0.05	0.18		
Dolichogenidea sp.	0.13		0.003			
Glyptapanteles sp.	0.03					
Microgastrinae sp.			0.01	0.03		
Parapanteles sp.	0.01					
Phaenocarpa sp.			0.0001			

Pholetesor sp.	0.002					
Protapanteles sp.	0.016					
Psyttalia humilis					0.004	
Psyttalia sp.					0.003	
Family: Chrysididae						
Chrysis subcoriacea		0.27				
Chrysis sp.		0.073			0.002	
Family: Colletidae						
Colletes sanctus			0.001			
Family: Dryinidae						
Aphelopus albifacialis			0.001			
Family: Eulophidae						
Neochrysocharis Formosa	0.27					
Family: Formicidae						
Anochetus graeffei	72.57	0.26	0.17			
Anochetus sp.	86.65	0.64	0.13		16.12	0.66
Anoplolepis gracilipes	0.33	0.2			0.05	0.52
Anoplolepis sp.					0.001	
Brachyponera croceicornis						0.11
Brachyponera obscurans			0.77	2.05	2.55	2.99
Brachyponera sp.			0.0003			
Brachyponera sp. 1						0.04
Brachyponera sp. 2						0.06
Brachyponera sp. 3						0.03

Camponotus sp.			0.001			0.15
Cardiocondyla minutior					0.003	
Cardiocondyla sp.					0.0004	
Carebara sp.						0.049
Chronoxenus sp.	0.009		0.004			
Colobopsis cylindrica		0.04				
Colobopsis saundersi		0.04				
Colobopsis sp.		1.304				
Crematogaster rogenhoferi			0	0.003		
Crematogaster sp.			0.01	0.02	0.02	
Dilobocondyla sp.	0.217					
Dolichoderus sp.			0.02			
Dolichoderus sp. 1					0.004	0.001
Dolichoderus sp. 2					0.004	0.001
Hypoponera punctatissima					0.003	
Hypoponera sp.			4.01	13.18		0.05
Hypoponera sp. 1						0.69
Hypoponera sp. 2						0.03
Hypoponera sp. 3					2.14	10.51
Hypoponera sp. 4						0.05
Hypoponera sp. 5						0.07
Hypoponera sp. 6					0.12	
Hypoponera sp. 7						0.05
Meranoplus bicolor			21.39	70.93	7.83	47.23
Meranoplus sp. 1					1.56	7.95
Meranoplus sp. 2						0.43
Meranoplus sp. 3					0.52	1.83

Meranoplus sp. 4					0.52	2.2
Meranoplus sp. 5						0.36
Meranoplus sp. 6						0.73
Myopopone sp.		0.489				
Nesomyrmex sp.			0.07			
Nylanderia sp.	0.02				0.0001	
Odontomachus simillimus		0.6	23.06	0.38		0.85
Odontomachus sp.		0.09	5.13	0.08		
Odontomachus sp. 1						0.11
Odontomachus sp. 2						0.004
Odontomachus sp. 3						0.003
Odontoponera sp.					0.02	
Oecophylla smaragdina	0.58					
Pheidole fervens					0.004	
Pheidole parva		0.04	8.61	0.5	0.4	7.18
Pheidole sauteri		0.14	28.51	1.65		
Pheidole sp.					0.05	0.59
Pheidole sp. 1	0.09	28.66				
Pheidole sp. 2	0.09	28.66				
Pheidole sp. 3	0.09					
Pheidole sp. 4	0.09					
Pheidole sp. 5	0.09					
Pheidole sp. 6	0.09					
Pheidole sp. 7	0.09					
Pheidole sp. 8		0.01				
Polyrhachis danum	0.04					
Polyrhachis lepida	0.08					
Polyrhachis sp.	0.05					
Prenolepis sp.	0.1					

Proatta butteli	4.76	1.24	1.28	3.87	82.87	2.02
Proatta sp.	0.85	0.34	1.04	3.82	66.34	2.54
Stictoponera bicolor	0.07					
Strumigenys emmae	1.55				0.02	0.1
Strumigenys membranifera					0.1	
Strumigenys sp.					0.05	
Strumigenys sp. 1					0.005	
Strumigenys sp. 2					0.001	
Tapinoma indicum					0.002	
Tapinoma melanocephalum			1.18		0.003	
Tapinoma sp.					0.005	
Tetramorium kydelphon						0.003
Tetramorium lanuginosum					0.03	
Tetramorium pacificum						0.03
Tetramorium sp.			0.1			
Vollenhovia sp.			0.42	1.62	0.27	0.76
Family: Ichneumonidae						
Acerataspis clavata	0.21		0.02		0.004	
Acerataspis sinensis	0.21		0.02		0.004	
Colaulus sp.			0.002			
Gessia sp.					0.004	
Mesochorus sp.	0.09			0.01		
Microcharops sp.	0.08					
Polyaulon sp.					0.004	
Stenomacrus sp.	0.02					

Xanthopimpla flavolineata			0.001	0.001	
Zonocryptus luctor					0.004
Zonocryptus sp.					0.003
Family: Megastigmidae					
Megastigmus sp.	0.03		0.004		
Megastigmus viggianii	0.17				
Family: Mymaridae					
Anagrus sp.	0.21				0.004
Family: Pteromalidae					
Anisopteromalus calandrae			0.001	0.001	
Order: Lepidoptera					
Family: Autostichidae					
Autosticha pachysticta		0.05			
Family: Bucculatricidae					
Bucculatrix sp.	0.02				
Family: Cosmopterigidae					
Macrobathra sp.		0.12			
Mimodoxa sp.	0.02				
Family: Crambidae					

Canuza sp. 1		0.07			
Glyphodes sp.		0.15			
Leucinodes orbonalis		0.01			
Leucinodes sp.					
Nevrina sp.		1.1			
Palpita sp.		0.14			
Parapoynx stagnalis		0.23	0.01		
Parapoynx villidalis				0.02	
Parapoynx sp.			0.02	0.03	
Scirpophaga incertulas					0.02
Scirpophaga sp. 1					0.001
Scirpophaga sp. 2					0.001
Sufetula sp.			0.03		
Family: Erebidae					
Asota sp.		0.03			
Bastilla sp.		0.05			
Cifuna sp.	0.02				
Euproctis sp.		0.03			
Hypospila sp.	0.02				
Family: Geometridae					
Chlorissa sp.	0.03				
Chrysocraspeda sp.		0.02		0.03	
Erannis sp.		0.02			
Scopula sp.					
Xanthorhoe sp.	0.02				

Family: Gracillariidae			
Caloptilia protiella		0.003	
Caloptilia sp.		0.01	
Phyllonorycter sp.			0.017
Family: Hesperiidae			
Burara sp.			0.017
Erionota acroleuca		0.02	
Erionota thrax		0.16	
Erionota sp.		0.063	
Synapte sp.		0.013	
Family: Limacodidae			
Phocoderma sp.		0.137	
Family: Lycaenidae			
Arhopala sp.		0.08	
Eirmocides sp.		0.009	
Jamides sp.		0.005	
Family: Noctuidae			
Elaphria sp.	0.339		
Helicoverpa sp.		0.028	
Heliocheilus sp.		0.088	
Schinia sp.		0.013	
Family: Nolidae			
Miaromima pangolina		0.05	
Miaromima sp.		0.015	
Nola sp.		0.027	

Pterogonia cardinalis		0.05		
Family: Nymphalidae				
Ypthima baldus		0.36		
Ypthima nebulosa		0.24		
Ypthima sp.		0.354		
Ypthimomorpha sp.		0.032		
Family: Papilionidae				
Graphium sp.		0.028		
Family: Pieridae				
Dercas sp.	0.023			
Family: Pyralidae				
Citripestis eutraphera			0.001	
Family: Sphingidae		0.038		
Agrius sp.	0.044			
Phylloxiphia sp.				
Family: Thyrididae				
Pyrinioides sp.				0.035
Family: Tineidae				
Erechthias zebrina			0.001	
Erechthias sp.			0.002	
Family: Tortricidae				
Epiblema sp.		0.059		

Homona sp.		0.028				
Olethreutes sp.		0.009				
Pandemis sp.		0.549				
Phaecasiophora sp.		0.118				
Spilonota sp.		0.059				
Sycacantha sp.		0.355				
Family: Xyloryctidae						
Scieropepla sp.	0.011	2.217			0.014	
Family: Ypsolophidae						
Ypsolopha sp.	0.023					
Family: Zygaenidae		0.55				
Eterusia sp.						
Order: Odonata						
Family: Aeshnidae						
Gynacantha Dravida			0.001			
Family: Coenagrionidae						
Agriocnemis femina			0.002			
Agriocnemis pygmaea			0.005			
Agriocnemis sp.			0.068			
Family: Libellulidae						
Diplacodes trivialis						0.02
Diplacodes sp.						0.02

Order: Orthroptera					
Family: Acrididae					
Dasyhippus barbipes			0.01		
Family: Gryllidae					
Teleogryllus occipitalis					0.10
Teleogryllus sp.					0.3
Order: Thysanoptera					
Family: Phlaeothripidae					
Cephalothrips sp.		0.001			
Cercyon sp.		0.002			
Haplothrips sp.	2.95				
Family: Thripidae					
Frankliniella occidentalis	1.67	0.15			
Frankliniella schultzei	0.42	0.04			
Frankliniella sp.	0.312	0.144			
Megalurothrips usitatus	0.09		0.01		
Megalurothrips sp.	0.014		0.005		
Scirtothrips dorsalis		0.003			
Scirtothrips sp.		0.004			
Thrips hawaiiensis				0.0001	
Thrips palmi				0.0001	
Order: Trichoptera					

Family: Hydropsychidae	
Potamyia sp.	0.009
